# cPRArgentina: a population-specific cPRA calculator incorporating optional ABO compatibility

**DOI:** 10.3389/fgene.2026.1971911

**Published:** 2026-10-19

**Authors:** Manuel Quirno Costa, María Belén Balekjian, María Soledad Paulos, María Emilia Bianco

**Affiliations:** 1 Oxford Transplant Centre, Churchill Hospital, Oxford University Hospitals NHS Foundation Trust, Oxford, United Kingdom; 2 Sección Histocompatibilidad, Laboratorio Central, Hospital General de Agudos “Carlos G. Durand”, Buenos Aires, Argentina; 3 Centro Provincial de Histocompatibilidad, Centro Único Coordinador de Ablación e Implante de La Provincia de Buenos Aires, La Plata, Argentina

**Keywords:** ABO compatibility, Argentina, cPRA, HLA, kidney transplantation, Latin America, organ allocation

## Abstract

Calculated panel reactive antibody (cPRA) is used to assess the proportion of donors expected to be incompatible with a transplant candidate based on unacceptable Human Leukocyte Antigen (HLA) specificities. Its accuracy depends on how well the reference donor population reflects the actual donor pool and on the compatibility criteria considered. We developed cPRArgentina, a web-based application that directly compares patient-specific unacceptable HLA antigens with deceased donor data from Argentina and can optionally incorporate ABO compatibility. We evaluated the application using 100 synthetic recipient profiles covering different levels of HLA sensitization and all ABO blood groups. Incorporating ABO compatibility increased cPRA in all blood groups except AB, with the greatest increases observed in groups O and B. Its effect was also greater among profiles with lower HLA-only cPRA and decreased as HLA sensitization increased. These findings show how incorporating ABO compatibility can change the picture of donor availability beyond HLA sensitization alone. cPRArgentina provides a population-specific approach to cPRA calculation using Argentine donor data, with a flexible design that can incorporate broader donor data and additional HLA information as they become available.

## 1 Introduction

Kidney transplantation remains the optimal treatment for end-stage renal disease (Wolfe et al., 1999), and successful organ allocation relies on accurate assessment of immunological compatibility between donors and recipients. The Human Leukocyte Antigen (HLA) system is central to this process, and sensitization to HLA antigens significantly reduces the likelihood of finding a compatible donor (Tambur et al., 2018).

Panel Reactive Antibody (PRA) has traditionally been used to estimate the degree of sensitization, representing the proportion of antigens in an assay panel against which a recipient shows antibody reactivity. Calculated PRA (cPRA) was introduced to estimate donor incompatibility based on unacceptable HLA antigens within a reference donor population (Cecka, 2010). The interpretation of both PRA and cPRA depends on how well the respective reference panel or donor population reflects the characteristics of donors available for transplantation (Alvares et al., 2023), and some models also incorporate clinically relevant criteria such as ABO compatibility, which directly influences access to transplantation (Gragert et al., 2022).

Latin American populations show substantial genetic diversity (Benirschke, 2002; Norris et al., 2018). HLA allele and haplotype distributions may differ from those observed in European and North American populations (Hurley et al., 2018; Fernández Souto et al., 2021), which have traditionally served as the basis for many commercial HLA antibody panels and reference donor datasets used by international cPRA calculators.

In Argentina, PRA assessment is primarily performed using commercial Luminex assays based on manufacturer-defined antigen panels. While these assays provide a standardized approach to PRA assessment, the antigen panels may not fully reflect the distribution of HLA antigens within the local donor population. They also do not account for ABO compatibility, a fundamental aspect of kidney transplantation that directly influences donor availability (Gragert et al., 2022).

To address this gap, we developed cPRArgentina, a web-based application that directly compares patient-specific unacceptable HLA antigens with donors in a reference dataset of deceased donors from the Province of Buenos Aires, Argentina. The application uses a donor-filtering approach and can optionally incorporate ABO compatibility. cPRArgentina was designed to allow the donor dataset to be updated as HLA data from other regions of Argentina, additional loci, or higher-resolution typing become available. In this work, we describe the implementation of the application and evaluate its behavior using simulated recipient profiles with different levels of HLA sensitization and ABO blood groups.

## 2 Methods

### 2.1 Data source

cPRArgentina uses HLA and ABO data from 4159 deceased kidney donors in the Province of Buenos Aires, Argentina, collected during routine clinical practice from 1998 to 2026 (Supplementary Table S1).

Serologic HLA-A, -B, and -DR assignments were available for all donors, whereas HLA-DQ typing was available for 1156 more recently typed donors. This reflects the historical evolution of HLA typing practices in Argentina, with HLA-DQ incorporated into routine donor typing from 2019 onwards.

### 2.2 cPRA calculation

User-provided unacceptable HLA antigens undergo nomenclature normalization and validation before calculation. Only supported split antigens are included in the analysis. Immunogenetically valid antigens that fall outside the current scope of the application are recognized and reported to the user but do not contribute to the calculation (Figure 1A). Likewise, broad antigens are recognized but excluded because split equivalents are not inferred (Figure 1B). Invalid entries prevent calculation until corrected (Figure 1C).

**FIGURE 1:**
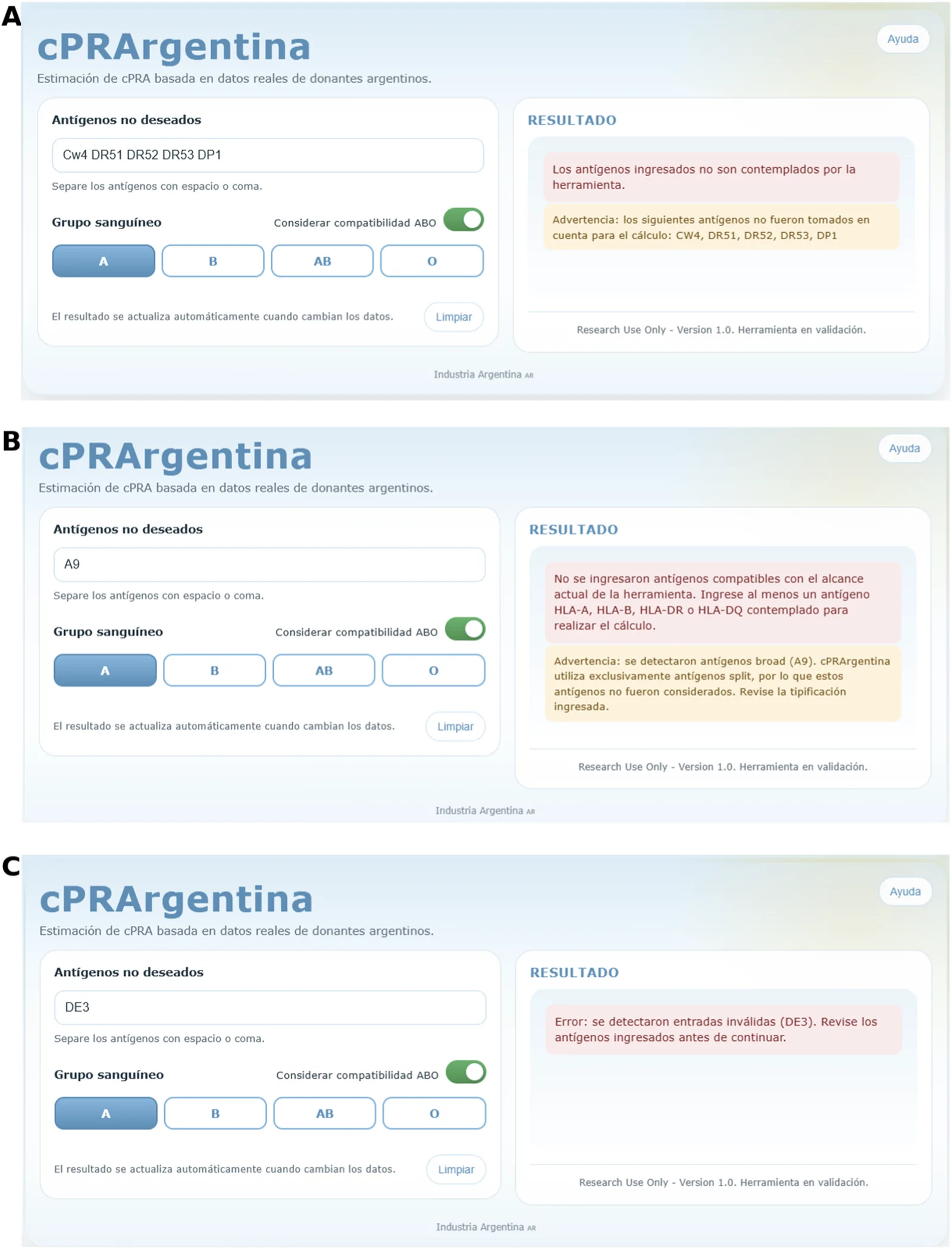
Examples of the validation process applied to user-entered unacceptable HLA antigens **(A)** Immunogenetically valid antigens outside the current scope of cPRArgentina are recognized and reported to the user but are not included in the calculation. **(B)** Broad antigens are recognized but excluded because split equivalents are not inferred. **(C)** Invalid entries generate a validation error and prevent calculation until corrected. (In each example, the left panel shows the unacceptable antigen entered by the user, while the right panel displays the corresponding validation message or warning).

After validation, each recipient’s unacceptable HLA antigens are directly compared with the HLA types of donors in the dataset. A donor is considered incompatible if at least one unacceptable antigen is present (Howell et al., 2010; Chan et al., 2017). The cPRA is then calculated as the proportion of incompatible donors among all donors included in the calculation:

$$cPRA=\frac{N_{\text{incompatible}\;\text{donors}}}{N_{\text{total}\;\text{donors}}}\;x100$$

All cPRA values are expressed as percentages, rounded to 1 decimal point, ranging from 0 to 100 (Figure 2).

**FIGURE 2:**
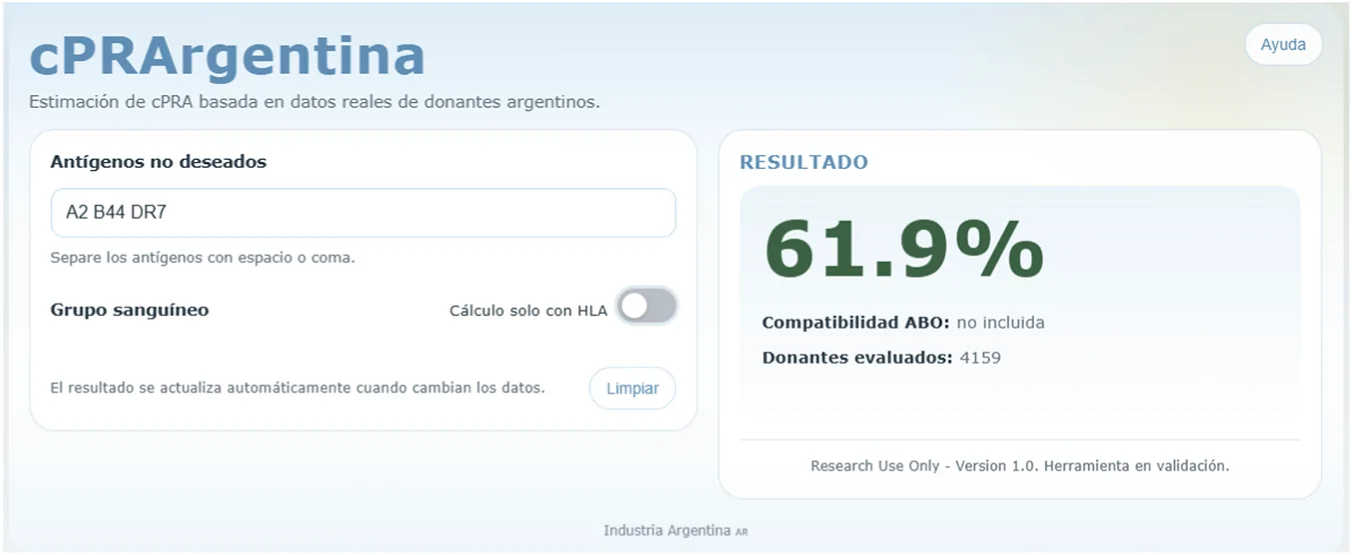
Example of the cPRArgentina interface showing user-entered unacceptable HLA antigens and the calculated cPRA result with ABO compatibility disabled (“HLA-only cPRA”) (The left panel shows the entered unacceptable antigens and the ABO compatibility toggle, while the right panel displays the calculated cPRA (61.9%), indicates that ABO compatibility is not included, and reports the number of donors evaluated (n = 4,159)).

### 2.3 Handling of HLA-DQ data

Because HLA-DQ typing was progressively incorporated into routine clinical practice, not all donor records include DQ information. To prevent underestimation of cPRA, the denominator is adjusted according to the unacceptable antigens entered by the user. All donors (n = 4159) are included when no HLA-DQ antigens are specified; however, if at least one supported DQ antigen is entered, the calculation is restricted to donors with complete HLA-A, -B, -DR, and -DQ typing (n = 1156) (Figure 3).

**FIGURE 3:**
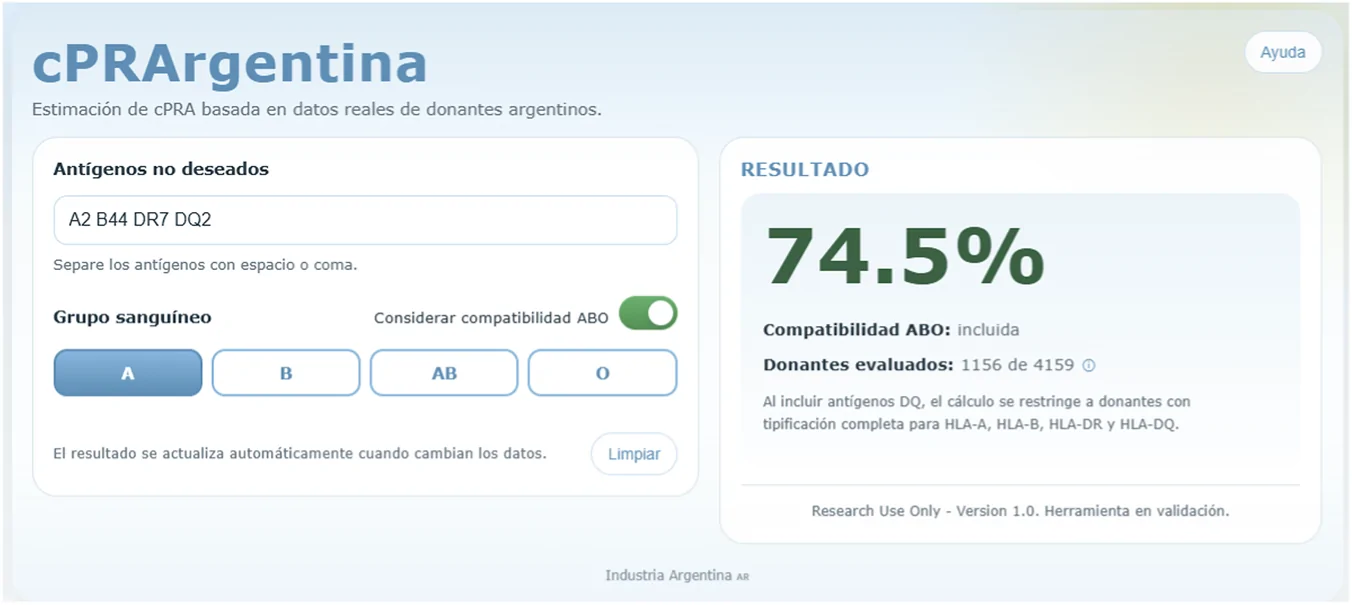
Example of cPRArgentina automatically adjusting the donor dataset when HLA-DQ antigens are included. The application restricts the calculation to donors with complete HLA-A, -B, -DR, and -DQ typing and reports the number of donors included in the calculation (The left panel shows a profile including DQ2 as an unacceptable antigen, while the right panel displays the calculated cPRA (74.5%) and informs the user that the calculation has been restricted to the 1,156 donors with complete HLA-A, -B, -DR, and -DQ typing.).

### 2.4 Incorporation of ABO compatibility

ABO compatibility can optionally be included in the cPRA calculation to better reflect donor availability. When this option is enabled, donors are considered incompatible if they meet either the HLA or ABO incompatibility criteria (Figure 4). When disabled, incompatibility is determined exclusively by HLA (“HLA-only cPRA”, Figure 2).

**FIGURE 4:**
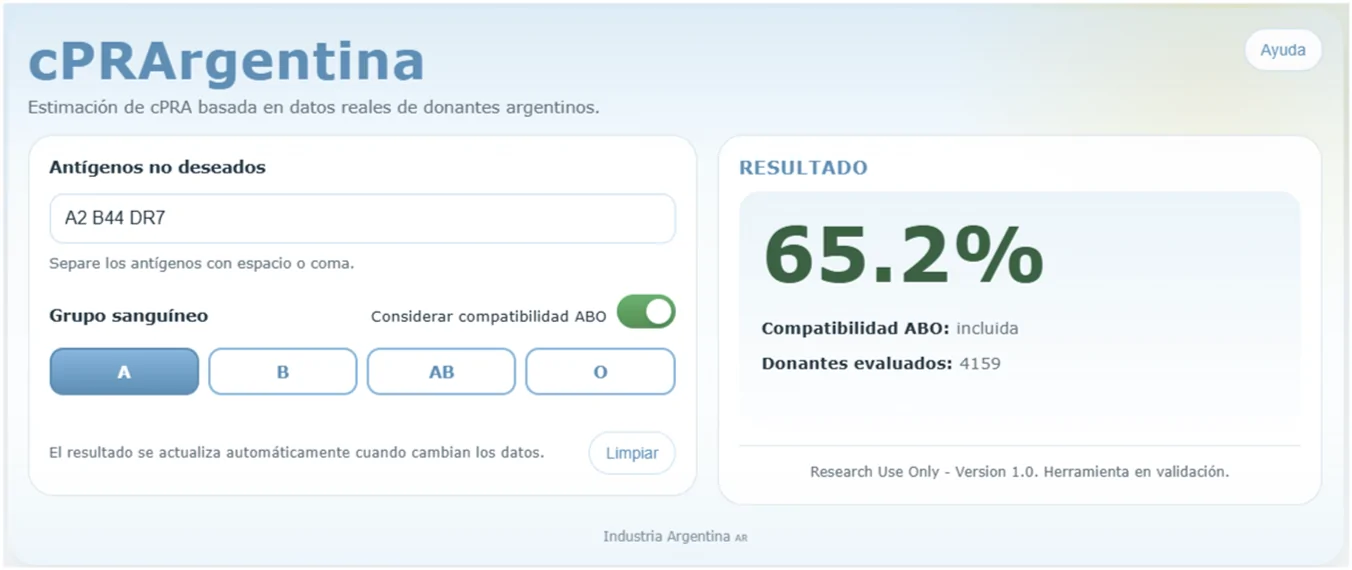
Example of the cPRArgentina interface showing user-entered unacceptable HLA antigens, recipient blood group selection, and the calculated cPRA result with ABO compatibility enabled (The left panel shows the entered unacceptable antigens, recipient blood group (A), and enabled ABO compatibility option, while the right panel displays the resulting cPRA (65.2%), confirms that ABO compatibility is included, and reports the number of donors evaluated (n = 4,159)).

The ABO-adjusted cPRA is therefore calculated as:

$$cPRA=\frac{N_{\left(HLA\cup ABO\right)\;\text{incompatible}\;\text{donors}}}{N_{\text{total}\;\text{donors}}}\;x\;100$$

The full donor pool was used as the denominator, so that cPRA represents the proportion of donors considered immunologically unacceptable based on HLA incompatibility and, when enabled, ABO incompatibility, following the approach described by Gragert et al. (2022).

### 2.5 Evaluation using simulated profiles

To evaluate the behavior of the application across different compatibility scenarios, 100 synthetic recipient profiles were generated, including recipients from all ABO blood groups and different levels of HLA sensitization (Supplementary Table S2). For each profile, cPRA was calculated both with and without ABO compatibility enabled.

For descriptive purposes, profiles were grouped according to their HLA-only cPRA values as low (<30%), intermediate (30%–69%), or high (≥70%). These categories were used only to summarize the simulated results and were not intended as clinical thresholds.

To assess the influence of the reference population on cPRA estimates, the same 100 synthetic profiles were evaluated using the Eurotransplant and OPTN calculators (accessed September 2026). HLA-only estimates were compared across the three calculators, while analyses incorporating ABO compatibility included Eurotransplant and the ABO-adjusted cPRA approach described by Gragert et al. (2022). As Eurotransplant reports ABO-compatible donor availability, its complementary value (100 − donor availability) was used to express results on the same incompatibility scale. Comparisons were descriptive and included Pearson correlations and mean absolute differences, overall and by sensitization category. The external calculators used for comparison were accessed in September 2026 and included the Eurotransplant vPRA calculator (https://www.etrl.org/vPRA.aspx), Eurotransplant ABO-compatible donor frequency calculator (https://www.etrl.org/FreqABOcompatible.aspx), OPTN cPRA calculator, and Gragert et al. ABO-adjusted cPRA calculator (https://transplanttoolbox.org/abo_hla_cpra/).

Results from all analyses are presented in the Results section.

### 2.6 Application implementation and testing

cPRArgentina was developed as a web-based application for cPRA calculation using Python and the FastAPI framework. Donor data are stored in an SQLite database, allowing real-time calculation from user-defined HLA and ABO inputs. The application is organized into separate components for the user interface, calculation logic, and data management.

The calculation workflow includes input nomenclature normalization, antigen validation, donor filtering, and cPRA calculation. Automated tests were implemented using pytest to verify key calculation steps and input validation. Formal code coverage assessment with pytest-cov showed that the automated test suite covered 75% of the code across the core backend modules and 100% of the central cPRA calculation module.

### 2.7 Software availability and reproducibility

cPRArgentina is publicly accessible as a web-based application at https://cprargentina.pythonanywhere.com/. The source code is available through the project’s GitHub repository (https://github.com/eme-cu-ce/cPRArgentina). Version 1.0.0, corresponding to the implementation evaluated in this work, has been archived in Zenodo and is available at DOI: 10.5281/zenodo.21893434.

The donor dataset used for the current implementation is provided in Supplementary Table S1, and the 100 simulated recipient profiles used for evaluation are provided in Supplementary Table S2. The simulated cohort is also encoded in the reproducibility script *scripts/run_cohort.py*, allowing the calculations reported in this work to be reproduced using the archived software version.

The software is available for research, educational, academic, and other non-commercial purposes. Commercial use requires prior written permission from the copyright holder.

## 3 Results

### 3.1 Evaluation scenarios

One hundred synthetic recipient profiles were used to evaluate the application across different compatibility scenarios. The profiles included recipients from all ABO blood groups, different levels of HLA sensitization, and scenarios with and without HLA-DQ antigens. The complete set of simulated profiles is provided in Supplementary Table S2.

### 3.2 Effect of incorporating ABO compatibility

Incorporating ABO compatibility increased cPRA compared with HLA-only cPRA in all recipient blood groups except AB. The increase was greatest among recipients with blood group O, followed by B, A, and AB (16.9, 15.1, 4.6, and 0 percentage points, respectively) (Figure 5).

**FIGURE 5:**
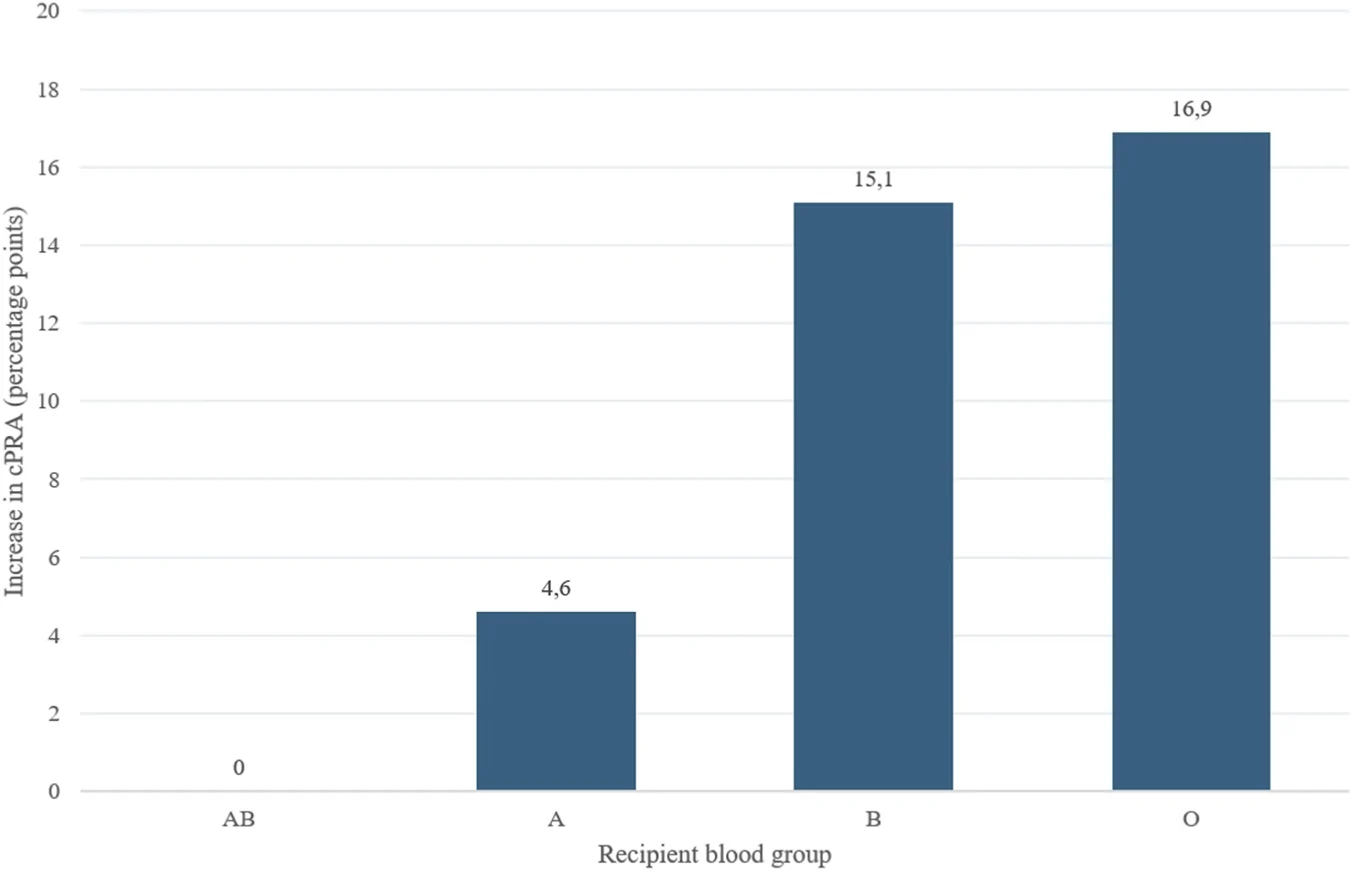
Increase in cPRA after incorporation of ABO compatibility according to recipient blood group. Bars represent the mean difference between cPRA calculated with ABO compatibility and HLA-only cPRA across the synthetic profiles within each group.

No increase was observed for recipients with blood group AB because all donor ABO groups were considered compatible (Table 1).

**TABLE 1:** ABO donor compatibility and blood group distribution in the donor dataset used for cPRA calculation.

Recipient blood group	Compatible donor blood groups	Donors by blood group, n (%)	Compatible donors, n (%)
O	O	2,403 (57.8%)	2,403 (57.8%)
A	A, O	1,353 (32.5%)	3,756 (90.4%)
B	B, O	312 (7.5%)	2,715 (65.3%)
AB	A, B, AB, O	91 (2.2%)	4,159 (100.0%)

### 3.3 Effect according to HLA sensitization level

The effect of ABO compatibility also varied according to HLA-only cPRA. The mean increase in cPRA was greatest among profiles with low sensitization, followed by intermediate and high sensitization (17.2, 13.4, and 6.5 percentage points, respectively) (Figure 6). Overall, the additional effect of ABO compatibility tended to decrease as HLA-only cPRA increased.

**FIGURE 6:**
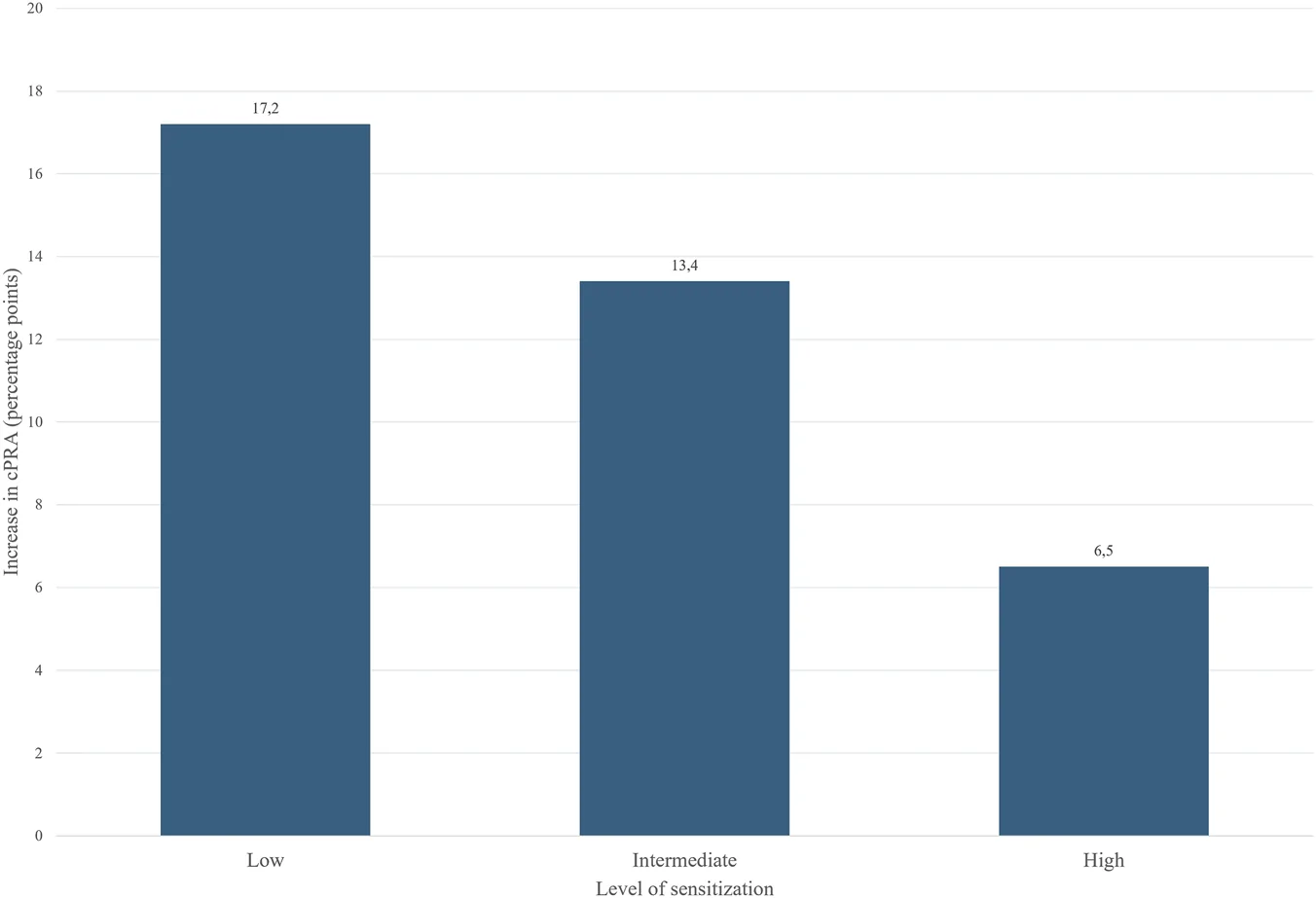
Increase in cPRA after incorporation of ABO compatibility according to baseline HLA sensitization level. Bars represent the mean difference between cPRA calculated with ABO compatibility and HLA-only cPRA across the synthetic profiles within each category.

### 3.4 Interaction between ABO group and HLA sensitization

When recipient ABO group and HLA sensitization were considered together, the largest increases were observed among group O and B recipients with lower HLA-only cPRA values (Supplementary Table S3). The impact of incorporating ABO compatibility depended on both recipient blood group and HLA sensitization.

### 3.5 Comparison with international cPRA calculators

HLA-only cPRA estimates showed high correlations between cPRArgentina and both Eurotransplant (r = 0.927) and OPTN (r = 0.958). Mean absolute differences were 6.71 and 4.36 percentage points, respectively, and were smaller among highly sensitized profiles than among low- and intermediate-sensitization profiles. A similar pattern was observed when ABO compatibility was incorporated, with correlations of 0.926 for Eurotransplant and 0.957 for the Gragert et al. ABO-adjusted cPRA approach. Mean absolute differences were 8.98 and 5.47 percentage points, respectively, and again decreased with increasing sensitization (Table 2). Individual profile results are provided in Supplementary Tables S4, S5.

**TABLE 2:** Comparison of cPRA estimates obtained with cPRArgentina and international calculators. Values for overall, low, intermediate, and high represent mean absolute differences in cPRA, expressed in percentage points. Sensitization categories were defined according to cPRArgentina HLA-only cPRA as low (<30%), intermediate (30%–69%), and high (≥70%). Pearson r represents the correlation across all 100 simulated profiles.

Comparison	Overall	Low	Intermediate	High	Pearson’s r
cPRArgentina vs. Eurotransplant, HLA-only	6.71	7.66	7.28	4.51	0.927
cPRArgentina vs. OPTN, HLA-only	4.36	5.39	4.79	2.58	0.958
cPRArgentina vs. Eurotransplant, HLA + ABO	8.98	13.46	9.67	5.01	0.926
cPRArgentina vs. Gragert et al., ABO-adjusted	5.47	8.86	5.96	2.58	0.957

### 3.6 Reproducibility

The analysis was repeated using cPRArgentina v1.0.0, and the same cPRA results were obtained for all 100 simulated profiles. Profiles including supported HLA-DQ antigens used the DQ-typed donor subset (n = 1156), while the remaining profiles used the complete donor dataset (n = 4159).

## 4 Discussion

International cPRA calculators are widely used to assess sensitized transplant candidates and support organ allocation in different countries. These approaches use different reference populations, ranging from deceased donor datasets to large stem cell donor registries (Tinckam et al., 2015; Kransdorf et al., 2021). However, these reference populations may not fully represent the characteristics of donors available in Argentina. Because donor HLA distributions vary among populations, the use of local data provides an opportunity to estimate cPRA using a reference population that more closely reflects the donors available for transplantation in that particular setting.

Our results show that including ABO compatibility increases cPRA, although the magnitude of this effect depends on both recipient blood group and HLA sensitization. As expected, recipients with blood group AB do not show an increase because all ABO donor groups are compatible with AB recipients, whereas recipients with blood groups O and B experience the greatest increases in cPRA. In addition, the contribution of ABO compatibility decreases as HLA-only cPRA increases, suggesting that the effect of ABO compatibility is greater when more HLA-compatible donors are available.

Although ABO compatibility is routinely considered during organ allocation, incorporating it directly into cPRA calculation provides a more realistic picture of donor availability.

During the initial development of cPRArgentina, cPRA values were compared with the OPTN and Eurotransplant calculators and showed high concordance (Quirno Costa et al., 2024). This comparison was performed using a smaller donor dataset and did not include ABO compatibility. In the present analysis, high correlations were again observed across calculators, although absolute differences remained and were more pronounced among profiles with low and intermediate sensitization. A similar pattern was observed when ABO compatibility was incorporated. These findings illustrate how cPRA estimates for the same antibody profile may vary when different reference populations and calculation approaches are used, highlighting the relevance of population-specific estimates in reflecting the donor pool of a particular setting.

In Argentina, the assessment of sensitization is still largely based on commercially available PRA screening assays using predefined antigen panels. Although these PRA kits provide useful information about the degree of sensitization, they provide limited information on individual HLA antibody specificities and do not estimate the proportion of incompatible donors within the local population. Single antigen bead (SAB) assays, however, could serve a dual purpose. They provide a more detailed antibody profile that can be used to define unacceptable HLA antigens, while the same information can also be used to calculate cPRA using a local donor dataset. This could reduce the need for separate commercial PRA screening assays.

This work has several limitations. First, the donor dataset represents deceased kidney donors from the Province of Buenos Aires and may not fully capture the diversity of the country. Although this district accounts for approximately 31% of deceased donor donation processes nationally, representing the largest contribution from any individual jurisdiction, a substantial proportion of the national donor population is not represented in the current dataset. Expanding the dataset to include donors from all regions could provide a more representative national reference population, while also allowing potential differences to be explored. This would help determine whether specific regional adjustments, like those incorporated in the Canadian cPRA calculator (Tinckam et al., 2015), are needed, or whether a single, unified national pool would adequately reflect the Argentine donor population.

Second, the current dataset predominantly includes serologic HLA-A, -B, and -DR typing, with HLA-DQ information available only for a subset of donors. This limits both the resolution and the number of HLA loci that can currently be included in the calculation. As more complete donor typing data become available, the dataset could be expanded to include additional loci and higher-resolution HLA information. Recent developments in HLA serologic classification could also be incorporated into future versions of the tool, including updated allele-to-serotype assignments (Osoegawa et al., 2024; Marsh et al., 2026).

Finally, the evaluation was performed using synthetic recipient profiles rather than real transplant candidates. Although these scenarios allowed us to evaluate the application under different compatibility conditions and compare estimates across calculators, validation in larger clinical cohorts will be necessary to determine its performance and potential utility in clinical practice.

In conclusion, cPRArgentina provides population-specific cPRA estimation using deceased kidney donor data from Argentina, with optional incorporation of ABO compatibility. This work represents an initial step toward adapting cPRA calculation to the Argentine donor population. As donor data from other regions, additional HLA information, and clinical validation become available, the tool can continue to evolve to better reflect donor availability for transplant candidates in Argentina.

## Data Availability

The original contributions presented in the study are included in the article/Supplementary Material, further inquiries can be directed to the corresponding author.
